# A comparative study on the clinical efficacy of percutaneous endoscopic lumbar discectomy and conventional open surgery in the treatment of lumbar disc herniation

**DOI:** 10.12669/pjms.40.3.6678

**Published:** 2024

**Authors:** Zheng Li, Chen-yang Jiang, Bao-shan Xu

**Affiliations:** 1 Zheng Li, Department of Orthopaedics, Tianjin Medical University, Tianjing 300203, China; 2 Chen-yang Jiang, Department of Orthopaedics, Baoding No.1 Hospital, Baoding, Hebei, 071000, P.R. China; 3 Bao-shan Xu, Department of Orthopaedics, Tianjin Medical University, Tianjing 300203, China

**Keywords:** Intervertebral disc displacement, Invasive surgical procedures, Treatment outcome

## Abstract

**Objective:**

To analyze the efficacy of single-channel percutaneous endoscopic lumbar discectomy (PELD) and conventional open surgery in the treatment of lumbar disc herniation (LDH).

**Methods:**

This is a retrospective study. A total of 66 patients with LDH admitted to Tianjin Medical University from June 2017 to June 2018 were divided into two groups: the observation group (single-channel PELD) and the control group (posterior lumbar interbody fusion), with 33 cases in each group. The two groups were compared in terms of visual analogue scale(VAS), oswestry disability index (ODI), Japanese Orthopaedic Association Score(JOA), perioperative indicators, clinical efficacy, postoperative complications, changes in inflammatory factors and serum T lymphocyte subsets.

**Results:**

The operation time, incision length, intraoperative blood loss, time in bed, hospital stay in the observation group were all lower than those in the control group. At 7d after treatment, the improvement of ODI, VAS and JOA in the observation group were better than that in the control group. At the last follow-up, there was no significant difference in Cobb angle and lumbar lordosis angle between the two groups. The levels of serum IL-1, IL-6 and TNF-α in the observation group were lower than those in the control group. The degree of reduction of serum CD3+ and CD4+ in the observation group were higher than those in the control group. And the level of elevation of CD8^+^ in the observation group was lower than that in the control group. Moreover, there was no significant difference in CD4+/CD8+ level between the two groups. The excellent rate of surgical results in the observation group was higher than that in the control group. Complications occurred in both groups, with no significant difference between the two groups.

**Conclusions:**

Single-channel PELD can achieve superior clinical efficacy over conventional open surgery in the treatment of LDH.

## INTRODUCTION

Lumbar disc herniation (LDH) is a common cause of pain in the lower back and legs clinically, which makes it difficult for patients to work and live normally, or to take care of themselves in severe cases.^1^,^2^ In clinical practice, conservative treatment and surgery are commonly used to treat this disease. Surgery includes percutaneous endoscopic lumbar discectomy (PDLE) and posterior lumbar interbody fusion (PLIF). PLIF is the current standard surgical method in clinical practice, boasting various advantages of high clinical cure rate and low recurrence rate. However, this surgical method causes great trauma, patients need to stay in bed for a long time after surgery, and are often accompanied by severe complications such as dural scar adhesion, and wound infection.^3^ With the development and optimization of minimally invasive endoscopy technology in recent years, the spinal endoscopic technique represented by percutaneous endoscopic lumbar discectomy (PELD) has been extensively applied in clinical practice and achieved remarkable therapeutic effects.^4^-^6^ Single-channel PELD is an extremely minimally invasive surgery with the advantages of local anesthesia, less trauma, no damage to the stable structure of the posterior column of the spine, and faster postoperative recovery compared with the traditional open surgery. In this study, a comparative analysis was performed on the efficacy of single-channel PELD for the treatment of LDH.

## METHODS

This is a retrospective study. A total of 66 patients with lumbar disc herniation(LDH) admitted to Tianjin Medical University from June 2017 to June 2018 were selected and divided into two groups: the observation group (single-channel percutaneous endoscopic lumbar discectomy) and the control group (posterior lumbar interbody fusion), with 33 cases in each group.

### Ethical Approval

The study was approved by the Institutional Ethics Committee of Tianjin Medical University (No.: 2022-037; March 28, 2022), and written informed consent was obtained from all participants.

### Inclusion criteria:

- Patients diagnosed with LDH by CT, MRI and other imaging examinations;^7^
- Patients with typical clinical manifestations such as lumbago and lower limb radialgia;
- Patients with nonsurgically treated responsibility segment;
- Patients with unsatisfactory conservative treatment for three months or more;
- Patients who themselves and their families know and agree to voluntarily undergo surgery and are willing to follow up regularly.

### Exclusion criteria:

- Patients with mental disorders and unable to communicate normally.
- Patients with severe osteoporosis, malignant tumors and severe infectious diseases.
- Patients with lumbar malformation and trauma.
- Patients with a history of lumbar surgery and contraindications to surgery.
- Patients with coronary heart disease, diabetes and other basic diseases.

Patients in the observation group received single-channel PELD. First, the puncture site was confirmed, the guide wire was inserted, the incision was made with a length of about 0.7 cm, and the subcutaneous channel was expanded. The working channel was inserted through the working channel. A foraminoscope was inserted through this channel, and the upper articular process of the lower vertebral body was excised with a trephine. The intervertebral foramen was enlarged, and the protruding and/or prolapsed nucleus pulposus was excised with a nucleus pulposus forceps in the spinal canal. After the nerve root relaxation was confirmed under the microscope, local irrigation was performed, the foraminoscope and working channel were removed, and the suture was performed after disinfection.

Patients in the control group underwent PLIF. A posterior median incision was made with the diseased segment as the center, and the skin, subcutaneous tissue and fascia were separated layer by layer to fully expose the decompression and fixation of the segment, pedicle screws were inserted, the lamina was opened, the intervertebral disc of the responsible segment was removed, and the endplate cartilage was scraped. Then, an appropriate interbody fusion device was selected for PLIF, and the screw rod was linked and fixed with pressure. After confirmation, the bleeding was stopped, the incision was cleaned, a drainage tube was placed, and the incision was sutured layer by layer. Used antibiotics to prevent infection after surgery, changed dressing and removed stitches on time, and guided patients in rehabilitation training.

### Observation indicators

All operations were performed by the same group of doctors.

###  Perioperative indicators

Operation time, incision length, intraoperative blood loss, postoperative ambulation time and length of hospital stay.

### Indicators related to lumbar function

The Cobb angle and lumbar lordosis angle were compared between the two groups before surgery and at the last follow-up. The oswestry disability index (ODI)^8^,^9^ and Japanese Orthopaedic Association Score (JOA) evaluation treatment score were compared between the two groups before surgery, 7d after surgery and at the last follow-up. A visual analogue scale (VAS) was used to assess the pain in both groups.^10^,^11^ Collected blood samples in all cases under fasting condition in the morning, T-lymphocyte subsets in peripheral blood were determined by flow cytometry. The levels of inflammatory factors (IL-1, IL-6 and TNF-α) and serum T lymphocyte subsets (CD3^+^, CD4^+^ and CD8^+^ and CD4^+^/CD8^+^) were observed before and after treatment in the two groups.

### Clinical efficacy

The clinical efficacy after half a year of treatment was evaluated with reference to the MacNab evaluation standard of lumbar spine function. Excellent: painless and able to move normally; Good: relieved symptoms, occasional pain, limited activity; Moderate: improved function, obvious pain, unable to perform normal activities; Poor: no change or aggravation of the condition. Excellent rate = excellent rate + good rate.

### Postoperative complications

The incidence of infection, nerve injury, thrombosis and dural injury in the two groups were counted.

### Statistical methods

SPSS 21.0 was used for statistical analysis of the data of the two groups. The measurement data was expressed as `x± s. An independent sample *t* test was utilized to compare the difference in operative data between the two groups, while paired sample *t* test was employed to compare preoperative and postoperative parameters of the same patient. c^2^ test was used to compare the incidence of complications between the two groups. A 95% confidence interval was used. P*<*0.05 indicates a statistically significant difference.

## RESULTS

There was no significant difference in preoperative general condition between the two groups (p>0.05), and it was comparable (Table-I). The operation time, time in bed and hospital stay of patients in the observation group were lower than those in the control group, and the incision length and blood loss were significantly lower than those in the control group (p<0.05), (Table-II).

**Table-I:** Comparison of general conditions between the two groups.

Group	n	Gender (number of cases)		Age (years old)	Follow-up time (months)	BMI	Lesion segment (number of cases)		
	
		Male	Female				L_3/4_	L_4/5_	L_5/S1_
Control group	33	20	13	61.64±9.72	31.09±8.22	26.61±2.70	4	23	6
Observation group	33	18	15	65.85±8.09	27.85±5.69	26.33±2.30	3	25	5
*t/c^2^* value		0.248		1.913	1.864	0.441	0.317		
*P* value		0.618		0.060	0.067	0.660	0.853		

**Table-II:** Comparison of perioperative conditions between the two groups (*χ̅*±s).

Group	Operation time (min)	Incision length (cm)	Blood loss (ml)	Time in bed (d)	Hospital stay (d)
Control group	115.15±6.67	7.32±0.58	91.97±8.00	7.46±1.03	14.42±1.48
Observation group	69.85±7.85	0.89±0.12	8.76±1.84	3.30±0.81	6.91±0.98
*t* value	25.253	61.877	58.249	18.166	24.326
*P* value	0.000	0.000	0.000	0.000	0.000

At the last follow-up after treatment, the Cobb gngle of the two groups was significantly lower than that before surgery, and the lumbar lordosis angle was significantly higher than that before surgery (p<0.05), with no statistically significant difference between the two groups (p>0.05), (Table-III).

**Table-III:** Comparison of lumbar spine related indicators between the two groups (*χ̅*±s).

Group	Cobb angle (°)		Lumbar lordosis angle (°)	

	Before surgery	Last follow-up	Before surgery	Last follow-up
Control group	20.18±1.51	10.76±0.12	25.18±1.10	36.15±1.23
Observation group	20.45±1.33	10.67±1.08	25.12±1.34	36.33±1.05
*t* value	0.780	0.336	0.201	0.646
*P* value	0.438	0.738	0.842	0.520

At seventh day after treatment and at the last follow-up, the ODI index and VAS score of the two groups were significantly lower than those before treatment, and the JOA score was significantly higher than that before treatment (p<0.05). At 7d after treatment, the improvement of ODI index, JOA and VAS scores in the observation group was better than that in the control group (p<0.05), but with no significant difference in the ODI index, JOA and VAS scores between the two groups at the last follow-up (p>0.05), (Table-IV).

**Table-IV:** Comparison of ODI index, JOA and VAS scores between the two groups (*χ̅*±s).

Group	ODI index			JOA score			VAS score		

	Before surgery	7d after surgery	Last follow-up	Before surgery	7d after surgery	Last follow-up	Before surgery	7d after surgery	Last follow-up
Control group	42.70±1.26	22.21±1.36	2.27±0.45	8.03±0.77	19.94±1.34	25.12±1.34	5.91±0.80	3.55±0.51	0.61±0.50
Observation group	42.55±1.37	21.39±1.09	2.15±0.36	7.61±1.00	20.76±1.23	25.18±1.10	6.21±0.60	2.21±0.42	0.45±0.51
*t* value	0.467	2.694	1.199	1.933	2.583	0.201	1.734	11.707	1.229
*P* value	0.642	0.009	0.235	0.058	0.012	0.842	0.088	0.000	0.224

At the last follow-up, the serum levels of IL-1, IL-6 and TNF-α in the two groups were lower than those before treatment, and the observation group was lower than the control group (p<0.05), (Table-V). At the last follow-up, the levels of serum CD3^+^ and CD4^+^ in the two groups were lower than those before treatment, and the degree of reduction in the observation group was higher than that in the control group (p<0.05). In addition, the level of CD8^+^ was higher than that before treatment, and the level of elevation in the observation group was lower than that in the control group (p<0.05). The levels of CD4^+^/CD8^+^ in the two groups were lower than those before treatment (p<0.05), but with no significant difference between the two groups (p>0.05), (Table-VI)

**Table-V:** Comparison of inflammatory factors between the two groups before and after treatment (*χ̅*±s, ng/mL).

Group	IL-1		IL-6		TNF-α	

	Before surgery	Last follow-up	Before surgery	Last follow-up	Before surgery	Last follow-up
Control group	11.87±0.82	5.76±0.82	16.66±1.33	9.00±1.34	27.90±1.20	10.47±2.10
Observation group	11.55±1.01	4.46±0.47	16.28±1.41	7.22±0.69	27.42±1.08	7.89±0.57
*t* value	1.430	7.897	1.102	6.798	1.682	6.837
*P* value	0.158	0.000	0.275	0.000	0.098	0.000

**Table-VI:** Comparison of the levels of serum T lymphocyte subsets before and after treatment in the two groups (*χ̅*±s).

Group	CD3^+^ (%)		CD4^+^ (%)		CD8^+^ (%)		CD4^+^/CD8^+^	

	Before surgery	Last follow-up	Before surgery	Last follow-up	Before surgery	Last follow-up	Before surgery	Last follow-up
Control group	63.02±7.25	48.84±7.04	57.67±4.40	38.48±5.06	21.56±4.06	28.88±4.60	2.74±0.34	1.37±0.33
Observation group	63.66±5.60	44.16±5.67	57.77±3.98	34.32±4.50	21.89±3.92	24.15±4.07	2.70±0.38	1.47±0.35
*t* value	0.407	2.974	0.100	3.535	0.342	4.425	0.376	1.117
*P* value	0.686	0.004	0.921	0.001	0.733	0.000	0.708	0.268

At the last follow-up after treatment, the excellent and good rates of surgical outcomes in the observation group were significantly higher than that in the control group (p<0.05), (Table-VII). There was no significant difference in the incidence of operative complications between the two groups. (p>0.05), (Table-VIII).

**Table-VII:** Comparison of the excellent and good rates of surgical outcomes between the two groups [*n*, (%)].

Group	n	Excellent	Good	Moderate	Poor	Excellent and good rates
Control group	33	19 (57.58)	5 (15.15)	7 (21.21)	2 (6.06)	24 (72.73)
Observation group	33	22 (66.67)	9 (27.27)	1 (3.03)	1 (3.03)	31 (93.94)
*c*^2^ value						5.345
*P* value						0.021

**Table-VIII:** Comparison of the incidence of operative complications between the two groups [*n*, (%)].

Group	n	Transient nerve injury	Dural tear	Nerve root injury	Intervertebral space infection	Lower extremity venous thrombosis	Total
Control group	33	2 (6.06)	2 (6.06)	1 (3.03)	0	0	5 (15.15)
Observation group	33	1 (3.03)	4 (12.12)	1 (3.03)	1 (3.03)	1 (3.03)	8 (24.24)
*c*^2^ value							0.862
*P* value							0.353

## DISCUSSION

In this study, the efficacy of clinical treatment in the observation group reached 93.94%, and there were significant differences between the control group and the observation indexes such as operation time, incision length, intraoperative blood loss, postoperative bed rest and hospital stay. No effect was caused on the lumbar Cobb angle and lordosis angle, and surgical complications were not significantly different from those in the control group. In addition, the ODI index, JOA and VAS scores of the two groups were significantly improved seventh day after the operation, and the observation group improved more significantly than the control group. This indicates that single-channel PELD has the advantages of a short operation time, less trauma, less intraoperative blood loss, and quick postoperative recovery. Its application in the treatment of LDH can improve the effectiveness, reliability and safety of clinical treatment.

With the development of society and changes in people’s living and working styles, LDH presents an increasing incidence and tends to make inroads in young people.^12^,^13^ PLIF is the most commonly used surgical method for LDH. However, this surgical method requires stripping the muscle tissue around the spine, which may destroy the medial branch of the spinal nerve, and leave chronic pain in the back and lumbar rigidity and other discomforts in the long term. Some patients even have to undergo surgery again. Spinal nerves, dural sac, and cauda equina are easily damaged when tissues such as lamina and intervertebral discs are removed during surgery.^14^ Not only that, this surgical method has little effect on extreme lateral and foraminal LDH.^15^ Patients need to stay in bed for a period of time after surgery, and are prone to complications such as hypostatic pneumonia, urinary tract infection, and lower extremity venous thrombosis, which limits the clinical application of this operation to a certain extent.

In recent years, single-channel PELD has become a new surgical method for LDH. Different from open surgery, single-channel PELD boasts that the lamina can be preserved without destroying the stability of the lumbar spine while ensuring the removal of the protruding nucleus pulposus and decompression. The surgical field of view can be expanded by means of a foraminal mirror, and the risks of dural sac injury, nerve tissue pulling, soft tissue stripping etc can be reduced. On the premise of ensuring effective clinical treatment, it can substantially reduce the occurrence of surgical complications.^16^

Pain in patients with LDH has a close bearing on the local inflammatory response and inflammatory stimulation. It has been pointed out in most studies that the pain of LDH patients will be significantly improved after the nucleus pulposus tissue is removed, and the local inflammatory response will also be reduced.^17^ According to the results of this study, the levels of IL-1, IL-6 and TNF-α in the two groups at the last follow-up were significantly lower than those before treatment, and the indexes of inflammatory factors in the observation group were significantly lower than those in the control group, suggesting that endoscopic foraminal surgery boasts less release of inflammatory mediators and a milder degree of the inflammatory response, thereby reducing postoperative pain and promoting rapid recovery of patients.

CD4^+^ T lymphocytes play a vital role in the autoimmune response induced by LDH. Tian et al.^18^ found that the levels of CD3^+^ and CD4^+^ T cells and the ratio of CD4^+^/CD8^+^ in the peripheral blood of LDH patients were higher than those of normal people, while the level of CD8^+^ T cells was lower than that of normal people. It was found in this study that the levels of serum CD3^+^ and CD4^+^ were lower than those before treatment at the last follow-up. The degree of decrease in the observation group was higher than that in the control group, while the level of CD8^+^ was increased compared with that before treatment, and the degree of elevation in the observation group was lower than that in the control group. In addition, the levels of CD4^+^/CD8^+^ were lower than those before treatment, with no statistically significant difference between the two groups.

## CONCLUSIONS

Single-channel PELD can achieve superior clinical efficacy over conventional open surgery in the treatment of LDH, boasting various advantages such as short operation time, small incision, less intraoperative blood loss, short time in bed, hospital stay, and no increase in surgical complications, which is worthy of clinical promotion.

### Authors’ Contributions:

**ZL:** Designed this study and prepared this manuscript.

**BX:** Collected and analyzed clinical data, and is responsible and accountable for the accuracy or integrity of the work.

**CJ:** Significantly revised this manuscript.
